# *Rickettsia typhi* as Cause of Fatal Encephalitic Typhus in Hospitalized Patients, Hamburg, Germany, 1940–1944

**DOI:** 10.3201/eid2411.171373

**Published:** 2018-11

**Authors:** Jessica Rauch, Birgit Muntau, Petra Eggert, Dennis Tappe

**Affiliations:** Bernhard Nocht Institute for Tropical Medicine, Hamburg, Germany

**Keywords:** *Rickettsia typhi*, *Rickettsia prowazekii*, rickettsiosis, typhus, World War II, body louse, flea, rat, nested PCR, Germany, encephalitic typhus, hospitalized patients, bacteria, vector-borne infections, T cells, CD4, CD8, immunohistochemistry, brain lesions, fatality, endemic typhus, murine typhus, typhus nodules, epidemic, outbreak

## Abstract

We evaluated formalin-fixed paraffin-embedded tissue specimens from 7 patients who died with encephalitic typhus in Hamburg, Germany, during World War II. The archived specimens included only central nervous system tissues >70 years old that had been stored at room temperature. We demonstrated successful detection of *Rickettsia typhi* DNA by a nested qPCR specific to *prsA* in 2 patients. These results indicate that *R. typhi* infections contributed to typhus outbreaks during World War II. Immunohistochemical analyses of brain tissue specimens of *R. typhi* DNA–positive and –negative specimens showed perivascular B-cell accumulation. Around blood vessels, nodular cell accumulations consisted of CD4-positive and CD8-positive T cells and CD68-positive microglia and macrophages; neutrophils were found rarely. These findings are similar to those of previously reported *R. prowazekii* tissue specimen testing. Because *R. typhi* and *R. prowazekii* infections can be clinically and histopathologically similar, molecular analyses should be performed to distinguish the 2 pathogens.

## Abstract 2

Clinical and histopathologic features of *R. prowazekii* and *R. typhi* typhus can be similar, so molecular analyses should be performed to distinguish the 2 pathogens.

*Rickettsia typhi* infection, also known as murine or endemic typhus, is, except for its often milder course, clinically indistinguishable from epidemic typhus caused by *R. prowazekii*. Infection with *R. typhi* or *R. prowazekii* causes a clinical syndrome of high fever, headache, and rash. The central nervous system (CNS), cardiac, and pulmonary complications that occur are responsible for fatality rates of 4% for untreated endemic typhus and 30% for epidemic typhus (*1**–**3*). Whereas *R. typhi* is transmitted by fleas (oriental rat flea *Xenopsylla cheopis* and cat flea *Ctenocephalides felis*), *R. prowazekii* is transmitted by the human body louse *Pediculus humanus corporis*. Both of these pathogens are obligate intracellular zoonotic bacteria and Biosafety Level 3 pathogens (*1**–**3*); *R. prowazekii* is classified as a Centers for Disease Control and Prevention category B bioweapon agent.

Human infection with these bacteria occurs after inoculation of flea or louse feces in the skin lesion caused by the arthropod bite or by inhalation of dust containing dried vector feces. The appearance of epidemic louseborne typhus is often attributed to overcrowding and unhygienic conditions, such as those seen in prisons and refugee, labor, and concentration camps, and is associated with poverty and war worldwide. In contrast, the occurrence of murine fleaborne typhus is sporadic and linked to the presence of rats, often in coastal subtropical regions. Large epidemics of louseborne typhus occurred during World War I and II, leading to high fatalities in civilian populations, forced laborers, imprisoned persons, and military personnel.

We examined brain tissue samples from persons who had died from typhus in an infectious disease hospital in Hamburg, Germany, during World War II. We characterized *R. typhi* and *R. prowazekii* infections by using histologic, immunohistochemical, and molecular techniques.

## Materials and Methods

### Typhus Cases

We identified typhus cases by screening the books of arrivals from the Bernhard Nocht Institute Department of Pathology (Hamburg) for clinical and histopathologic descriptions of typhus. The Bernhard Nocht Institute Department of Pathology served as a center for infectious disease pathology diagnosis and received typhus specimens from multiple hospitals in Hamburg. We retrieved from the archives formalin-fixed, paraffin-embedded (FFPE) tissue blocks, which had been stored at room temperature. Clearance by the local ethics committee was obtained (no. WF-034/17) for our analyses.

### Histology and Immunohistochemical Analyses

For each FFPE tissue block, we analyzed a standard hematoxylin and eosin stained section microscopically for typhus nodules and documented the presence and numbers of lesions semiquantitatively. We screened sections for intracellular rickettsiae using Giemsa stains.

We performed immunohistochemical studies with antibodies against CD3 (1:400 dilution; EpitMics, Burlingame, CA, USA), CD20 (1:150 dilution; Agilent, Santa Clara, CA, USA), CD4 (1:30 dilution; Cell Marque, Rocklin, CA, USA), CD8 (1:20 dilution; Cell Marque), CD68 (1:100 dilution; Agilent), CD177 (1:33 dilution; Zytomed, Berlin, Germany), and inducible nitric oxide synthase (iNOS, 1:100 dilution; Zytomed). After pretreatment of FFPE tissue sections with buffers containing Trilogy (medac diagnostika, Tornesch, Germany; at 95°C for CD177), EDTA (pH 8 for CD4), or citrate (pH 6 for CD3, CD20, CD8, CD68, and iNOS) and endogenous peroxidase blocking, we incubated the sections with the respective antibodies in Antibody Diluent Solution (Zytomed) at 4°C overnight. Then, we incubated with either AEC 2-Component Detection Kit and 3-amino-9-ethylcarbazole substrate (DCS, Hamburg, Germany) for immunoperoxidase staining or AP Detection Kit and Fast Blue substrate (DCS) for immunophosphatase staining. Brain tissue from 5 patients without encephalitis served as negative controls, and lymphatic tissue served as a positive control for immunologic staining of immune cells.

### Molecular Assays

We ran samples through 3 rounds of processing: FFPE tissue block sectioning, DNA extraction, and quantitative PCR (qPCR). For each round, FFPE tissue blocks from typhus patients and negative control patients (patients with unrelated conditions, e.g., liver amebiasis) were placed in alternating order (i.e., 2 typhus patient samples, 1 negative control, 2 typhus patient samples, 1 negative control, and so on), cut into 5-µm slices, and deparaffinized. Before and after each round of sectioning and between sectioning different samples, we cleaned the microtome and microtome blades with DNA-ExitusPlus (AppliChem, Darmstadt, Germany). We performed DNA extractions with tissue sections in the same order as previously mentioned using the QIAamp DNA FFPE Tissue Kit (QIAGEN, Hilden, Germany). We included 2 additional negative controls (water) per round of extraction.

We performed panrickettsial real-time qPCRs targeting the *ompB* and *gltA* genes (*4**,**5*). In addition, we used a typhus group rickettsiae–specific qPCR targeting the *rpr331* gene (*6*) and nested species-specific qPCRs detecting the *prsA* genes (*7**,**8*) of the *R. typhi* and *R. prowazekii* genomes (Technical Appendix Table). We ran a β-actin gene qPCR (*9*) in parallel to check DNA quality.

Tissue sectioning, DNA extraction, and qPCR were performed in different levels of the building and by different personnel. No positive control was used in the qPCR.

## Results

Seven patients (1 in 1940 and 6 in 1944) had a clinical diagnosis of typhus and a histopathologic diagnosis of typhus on the basis of FFPE tissue blocks. All samples were of CNS tissues (cerebral cortex, pons, medulla oblongata, and cerebellum); 1–3 different CNS regions were available per patient (Table). All but the case from 1940 (patient 6) originated from Langenhorn hospital in the northern part of Hamburg.

**Table:** Characteristics and results of microscopic and molecular analyses of patients with typhus during World War II, Hamburg, Germany, 1940–1944*

Patient no.	Age, y/sex	Brain region	Frequency of typhus nodules	β-actin qPCR, C_t_, round 1/2/3†	*Rickettsia typhi*-specific nested *prsA* qPCR, C_t_
1	U/U	Medulla	+	37.2/–/44.5	–
		Pons	+++	–/–/35.7	–
2	U/U	Cerebellum	+	39.1/37.3/39.6	–
		Pons	++	37.1/34.7/37.2	–
		Cortex	++	35.3/33.3/38.1	–
3	U/U	Pons	++	37.3/–/**38.1**	23.8
		Cortex	+	35.1/–/37.7	–
4	U/U	Medulla	+++	–/–/42.5	–
		Pons	+	37.8/–/35.4	–
5	U/M	Pons	++	–/35.9/–	–
		Pons	+	**31.0/**–/–	32.4
6	U/U	Medulla	+	36.9/–/–	–
7	29/F	Cerebellum	+	40.0/35.1/–	–

*C_t_, cycle threshold; qPCR, quantitative PCR; U, unknown; +, ++, +++, semiquantitative microscopic measurements (+, low; ++, medium; +++, high); –, negative result. †From each brain region block, three 5-µm tissue sections were used for each round (n = 3) of extraction and amplification. The β-actin qPCR C_t_ of the respective round from which successful *R. typhi* DNA amplification was achieved is bolded.

**Figure 1:**
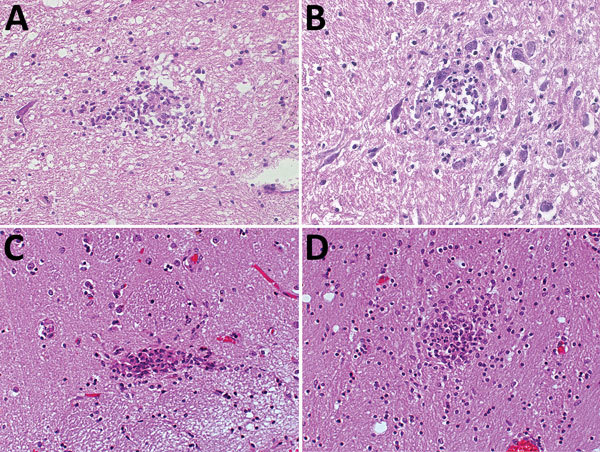
Hematoxylin and eosin staining of typical typhus nodules in brain of typhus patients during World War II, Hamburg, Germany, 1940–1944. Most nodules were found in the pons and medulla oblongata. A) Loose nodule. B) Spongy nodule amid large neuronal cells. C) Compact typhus nodule along longitudinal blood vessel. Note hyperemia of other blood vessels nearby. D) Another compact nodule with hyperemic blood vessels nearby. Original magnifications ×40.

**Figure 2:**
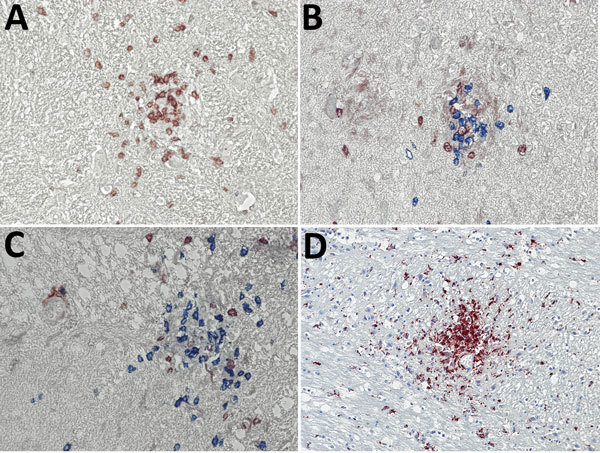
Immunohistochemical analyses of nodule cell compositions from typhus patients during World War II, Hamburg, Germany, 1940–1944. Tissue sections were incubated with specific antibodies and visualized with immunoperoxidase (brown) or immunophosphatase (blue) stains and lightly counterstained with hematoxylin. A) CD3 stain (brown) for T cells and CD20 stain (blue) for B cells. Only T cells are visible within the nodule. Original magnification ×40. B, C) CD4 stain (brown) and CD8 stain (blue) for T cell subsets. The nodules consist of a mixture of both cell types, with a predominance of CD8-positive cytotoxic T cells. Original magnifications ×40. D) CD68 stain (brown) for macrophages and microglia. A strong positivity is seen in the nodules and staining is also scattered in the surrounding brain parenchyma. Original magnification ×20.

Typical typhus nodules (Figure 1) were found in all cases. Lesions varied in size and location; most were seen in the pons or medulla oblongata (Table). No intracellular rickettsiae were found in Giemsa-stained sections. Immunohistochemical stains with CD20 antibody indicated B cells were only rarely present in perivascular regions and were not found in nodules. In contrast, T cells (CD3-positive cells) contributed prominently to nodule cell composition, consisting of ≈60% CD8+ T cells and ≈40% CD4+ T cells (Figure 2, panels A–C). CD177+ neutrophils were found in nodules rarely, and when present, these neutrophils were scattered mostly throughout the brain parenchyma (D. Tappe, unpub. data). In contrast, a strong signal was seen for CD68+ microglia and macrophages in and around the nodules (Figure 2, panel D). No iNOS+ cells were found (D. Tappe, unpub. data).

The panrickettsial qPCR, typhus group rickettsiae–specific *rpr331* qPCR, and *R. prowazekii*–specific nested qPCR results indicated all samples were negative for rickettsial genomic material. However, the nested *prsA* qPCR indicated tissue sections of the pons of 2 patients (patient 3 and patient 5; Table) were positive for *R. typhi* DNA. Positive qPCR results were obtained again with the pons of these patients when the extractions and qPCRs were repeated. All negative controls (FFPE blocks from patients with unrelated conditions and water) were negative. We sequenced the 2 amplicons (201 bp in length, including sequences of the outer primers; patient 3 GenBank accession no. MH553441), and BLAST analyses (http://www.blast.ncbi.nlm.nih.gov) showed sequence identity with *R. typhi* strains B9991CWPP (GenBank accession no. CP003398), TH1527 (GenBank accession no. CP003397), and Wilmington (GenBank accession no. AE017197) (Technical Appendix Figure).

## Discussion

Cases of typhus resulting from war, population displacement, poverty, and overcrowding are usually attributed to epidemic louseborne *R. prowazekii* (*1*). We show successful amplification of *R. typhi* DNA for 2 out of 7 patients with fatal typhus and encephalitis during World War II by a specific nested qPCR with FFPE CNS tissue samples stored for >70 years. Because of this finding, the diagnosis of murine typhus was made retrospectively for these 2 patients. For the remaining 5 patients, no rickettsial DNA could be amplified in the limited tissue materials available, possibly because of poor DNA quality. Nested PCRs are more sensitive than conventional PCRs, which could explain why the panrickettsial qPCRs and typhus group–specific qPCRs did not amply genomic DNA, whereas the nested PCR did with samples from 2 patients. Because nested PCRs are prone to contamination, we took several precautions: we included several negative controls (which remained negative throughout the study), omitted the positive control, and ran the nested PCR as a qPCR.

No intracellular bacteria were found by conventional light microscopy in Giemsa-stained brain sections. We could only assess CNS tissues because other tissue types had not been archived; the reason for the absence of these tissues is unclear. Microscopy results negative for rickettsial bacteria in brain tissue sections of typhus patients are in line with old reports; the doubtless detection of rickettsiae on microscopic examination of epidemic typhus patient samples was notoriously difficult (*10**,**11*). We did not attempt immunofluorescence testing of tissue sections. The postmortem detection of tissue nodules in brain sections during microscopic examination has been regarded as specific for epidemic typhus. Such typhus nodules were first described in the skin of patients with rash during epidemic typhus (typhus exanthematicus) in 1913. Shortly thereafter, during World War I, these nodules were found postmortem in the brains of epidemic typhus patients. The nodules were shown to be most prominent in the medulla and pons, also as seen in our study, followed by the basal ganglia, cortex, hippocampus, and cerebellum (*11**–**13*). In the 1910s, typhus was correctly described as a discontinuous vasculitis that could also occur in the brain involving lesions that form in endothelial cells of the intima of capillaries and small arteries. Inflammatory nodules always arranged around a blood vessel and were composed of lymphocytes, plasma cells, neutrophil granulocytes, and glia cells (*12**,**13*). In contrast with autopsy data of epidemic typhus, only rare reports of histopathologic findings of murine typhus exist. Typical rickettsial vascular injury and perivasculitis with lymphocytes, monocytes, and macrophages had been described in the CNS of murine typhus cases (*14**,**15*). In our study, immunohistochemistry confirmed the histopathologic findings of all these old reports. We also differentiated the T-cell subsets, showing that most T cells are CD8 positive. Using histopathology and immunohistochemistry, we found no obvious cell composition differences between nodules positive and those negative for *R. typhi* DNA. Thus, a similar histologic architecture and composition of typhus nodules in murine and epidemic typhus can be assumed.

The 7 cases examined in our study represent a minor fraction of the typhus cases seen in Langenhorn Hospital. The hospital had opened a ward specifically for typhus patients in May 1942, and 320 cases were seen during the following 2 years (*16*). Forced laborers from Russia who had typhus had also been admitted to this hospital (*17*).

Murine typhus is often a mild illness but can become more severe in refugee camps (*18*), and fatal and severe CNS cases of murine typhus have been described (*14**,**19**,**20*). Murine and epidemic typhus can be discerned neither histopathologically nor clinically (except perhaps by their expected respective severities and associated mortality rates, the presence of vectors, or flea or louse bite reactions on the skin). Eschars are usually absent in both forms of typhus. Serologic discrimination of the 2 species by cross-absorption and Western blotting (*21*) and molecular differentiation are confined to reference laboratories and only became available half a century after the cases were investigated.

The natural reservoir for *R. typhi* is rats, and unhygienic conditions that occur during times of civil disturbance promote the expansion of rat populations and their fleas and, thus, also the likelihood of persons acquiring murine typhus. Fleas remain infected for life, and their lifespan and feeding behavior are not altered by *R. typhi* infection (*22*). However, the *R. typhi* bacterium has also been shown experimentally to replicate in the body louse (*22*). Similar to infection with *R. prowazekii*, lice infected with *R. typhi* become red due to the rupture of their epithelial gut linings caused by the multiplication of rickettsiae, which reduces their lifespan. Thus, we speculate that *R. typhi* might also be transmitted by body lice under conditions of civil disturbance previously shown to favor the spread of *R. prowazekii*.

In conclusion, we show evidence that *R. typhi* played a role during local outbreaks or epidemics of typhus that were classically attributed to *R. prowazekii*. Therefore, more cases of typhus should be investigated molecularly to determine the type of rickettsial pathogen involved. Whether *R. typhi* was transmitted during World War II by lice remains to be elucidated.

Technical Appendix Primer and probe information and CLUSTAL O sequence alignment of *prsA* gene in phylogenetic analysis of samples from typhus patients during World War II, Hamburg, Germany, 1940–1944.
