# Supplementary material for: Rickettsia typhi as Cause of Fatal Encephalitic Typhus in Hospitalized Patients, Hamburg, Germany, 1940–1944
Source: Emerg Infect Dis. 2018 Nov;24(11):1982–7. doi: 10.3201/eid2411.171373 (PMC6200005; doi:10.3201/eid2411.171373)
Supplement: Technical Appendix — Primer and probe information and CLUSTAL O sequence alignment of prsA gene in phylogenetic analysis of samples from typhus patients during World War II, Hamburg, Germany, 1940–1944. [file 17-1373-Techapp-s1.pdf]

Article DOI: https://doi.org/10.3201/eid2411.171373

Rickettsia typhi as Cause of Fatal Encephalitic Typhus in Hospitalized Patients, Hamburg, Germany, 1940–1944

Technical Appendix

Technical Appendix Table. Information on primer and probes used for real-time quantitative PCR of *Rickettsia typhi prsA* gene*

PCR primer and probes	Sequence	Primer positioning
R. typhi strain Wilmington AE017197.1 (*1*)		
Outer-F	5′-GCTTGCAGAAGAATTCTCACTTG-3′	733310-733332
Outer-R	5′-GG**TA**CAGGTTTTTTCTCAAGCAC-3′	733488–733510
Inner-F for nested PCR	5′-ACAGCT**T**CAAATGGTGGGGT-3′	733341–733360
Inner-R for nested PCR	5′-TGCCAGCCGAAA**TC**TGTTTTG-3′	733457–733477
R. prowazekii strain Naples-1 CP014865.1 (*2*)		
Outer-F	5′-GCTTGCAGAAGAATTCTCTCTTG-3′	726697–726719
Outer-R	5′-GG**C**ACAGGTTTTTTTCAAGCAC-3′	726897–726875
Inner-F for nested PCR	5′-CAGCGTCAAATGGTGGG**A**TT-3′	726729–726746
Inner-R for nested PCR	5′-TGCCA**A**CCGAAA**CT**TGTTTTG-3′	726864–726844
Probe (*2*)		
R. typhi	5′-ATCAA**T**CAGGGCA**A**TTAGTACCAGAA-3′	733381-733406
R. prowazeki	5′-ATCAA**C**CAGGGCA**G**TTAGTACCAGAA-3′	726768-726793

*Nucleotides that differ between *R. typhi* and *R. prowazekii* DNA are highlighted in boldface.

```
CP003398_R.typhi          gcttgcagaagaattctcacttgataaagctacagcttcaaatggtggggttataggtta   60
CP003397_R.typhi          gcttgcagaagaattctcacttgataaagctacagcttcaaatggtggggttataggtta   60
AE017197_R.typhi          gcttgcagaagaattctcacttgataaagctacagcttcaaatggtggggttataggtta   60
Patient 3                 gcttgcagaagaattctcacttgataaagctacagcttcaaatggtggggttataggtta   60
Patient 5                 gcttgcagaagaattctcacttgataaagctacagcttcaaatggtggggttataggtta   60
CP014865_R.prowazekii     gcttgcagaagaattctctcttgataaagctacagcgtcaaatggtgggattataggtta   60

CP003398_R.typhi          cattatactaaatcaatcagggcaattagtaccagaatttgaaaataaagcgtttgcatt   120
CP003397_R.typhi          cattatactaaatcaatcagggcaattagtaccagaatttgaaaataaagcgtttgcatt   120
AE017197_R.typhi          cattatactaaatcaatcagggcaattagtaccagaatttgaaaataaagcgtttgcatt   120
Patient 3                 cattatactaaatcaatcagggcaattagtaccagaatttgaaaataaagcgtttgcatt   120
Patient 5                 cattatactaaatcaatcagggcaattagtaccagaatttgaaaataaagcgtttgcatt   120
CP014865_R.prowazekii     tattatactaaatcaaccagggcagttagtaccagaatttgaacagaaagcttttgcatt   120

CP003398_R.typhi          aaaagtaaatgaagtgtcaactccagtcaaaacagatttcggctggcatattataaaagt   180
CP003397_R.typhi          aaaagtaaatgaagtgtcaactccagtcaaaacagatttcggctggcatattataaaagt   180
AE017197_R.typhi          aaaagtaaatgaagtgtcaactccagtcaaaacagatttcggctggcatattataaaagt   180
Patient 3                 aaaagtaaatgaagtgtcaactccagtcaaaacagatttcggctggcatattataaaagt   180
Patient 5                 aaaagtaaatgaagtgtcaactccagtcaaaacagatttcggctggcatattataaaagt   180
CP014865_R.prowazekii     aaaagtaaatgaagtttcaactccagtcaaaacaagtttcggttggcatattataaaagt   180

CP003398_R.typhi          gcttgagaaaaaacctgtacc     201
CP003397_R.typhi          gcttgagaaaaaacctgtacc     201
AE017197_R.typhi          gcttgagaaaaaacctgtacc     201
Patient 3                 gcttgagaaaaaacctgtacc     201
Patient 5                 gcttgagaaaaaacctgtacc     201
CP014865_R.prowazekii     gcttgaaaaaaaacctgtgcc     201
```

Technical Appendix Figure. CLUSTAL O (1.2.4) multiple sequence alignment of partial *prsA* gene from *Rickettsia typhi* and *R. prowazekii* reference strains and bacteria from 2 patients hospitalized with typhus during World War II, Hamburg, Germany, 1940–1944. GenBank accession nos. of reference strains are

provided in alignment. The nucleotides that differ between the *R. prowazekii* sequence and *R. typhi* sequences are highlighted. Sequences of bacteria from patients 3 and 5 are identical to *R. typhi* rickettsia.

References

1. Papp S, Rauch J, Kuehl S, Richardt U, Keller C, Osterloh A. Comparative evaluation of two Rickettsia typhi-specific quantitative real-time PCRs for research and diagnostic purposes. Med Microbiol Immunol (Berl). 2017;206:41–51. PubMed http://dx.doi.org/10.1007/s00430-016-0480-z

2. Rauch J, Eisermann P, Noack B, Mehlhoop U, Muntau B, Schäfer J, et al. Typhus Group Rickettsiosis, Germany, 2010-2017. Emerg Infect Dis. 2018;24:1213–20. PubMed http://dx.doi.org/10.3201/eid2407.180093
